# Structure-based classification and ontology in chemistry

**DOI:** 10.1186/1758-2946-4-8

**Published:** 2012-04-05

**Authors:** Janna Hastings, Despoina Magka, Colin Batchelor, Lian Duan, Robert Stevens, Marcus Ennis, Christoph Steinbeck

**Affiliations:** 1 Cheminformatics and Metabolism, European Bioinformatics Institute, Hinxton, UK; 2 Swiss Center for Affective Sciences, University of Geneva, Geneva, Switzerland; 3 Department of Computer Science, University of Oxford, Oxford, UK; 4 Royal Society of Chemistry, Cambridge, UK; 5 ETH, Zürich, Switzerland; 6 School of Computer Science, University of Manchester, Manchester, UK

## Abstract

**Background:**

Recent years have seen an explosion in the availability of data in the chemistry domain. With this information explosion, however, retrieving *relevant *results from the available information, and *organising *those results, become even harder problems. Computational processing is essential to filter and organise the available resources so as to better facilitate the work of scientists. Ontologies encode expert domain knowledge in a hierarchically organised machine-processable format. One such ontology for the chemical domain is ChEBI. ChEBI provides a classification of chemicals based on their structural features and a role or activity-based classification. An example of a structure-based class is 'pentacyclic compound' (compounds containing five-ring structures), while an example of a role-based class is 'analgesic', since many different chemicals can act as analgesics without sharing structural features. Structure-based classification in chemistry exploits elegant regularities and symmetries in the underlying chemical domain. As yet, there has been neither a systematic analysis of the types of structural classification in use in chemistry nor a comparison to the capabilities of available technologies.

**Results:**

We analyze the different categories of structural classes in chemistry, presenting a list of patterns for features found in class definitions. We compare these patterns of class definition to tools which allow for automation of hierarchy construction within cheminformatics and within logic-based ontology technology, going into detail in the latter case with respect to the expressive capabilities of the Web Ontology Language and recent extensions for modelling structured objects. Finally we discuss the relationships and interactions between cheminformatics approaches and logic-based approaches.

**Conclusion:**

Systems that perform intelligent reasoning tasks on chemistry data require a diverse set of underlying computational utilities including algorithmic, statistical and logic-based tools. For the task of automatic structure-based classification of chemical entities, essential to managing the vast swathes of chemical data being brought online, systems which are capable of hybrid reasoning combining several different approaches are crucial. We provide a thorough review of the available tools and methodologies, and identify areas of open research.

## Background

Recent years have seen an explosion in the availability of data throughout the natural sciences. Availability of data facilitates research through complex data-mining and knowledge discovery methods. However, with the information explosion, retrieving *relevant *information from these data has become much more difficult. Computational processing is essential to filter, retrieve and organise such data. Traditional large-scale data management methods in chemistry include chemical structure-based algorithmic and statistical methods for the construction of hierarchies and similarity landscapes. These techniques are essential not only for human consumption of data in the form of effective browsing and searching but also in scientific methods for interpreting underlying biological mechanisms and detecting bioactivity patterns associated with chemical structure [1].

In biomedicine and the natural sciences more generally, hierarchical organisation and large-scale data management are being facilitated by *formal ontologies*: machine-understandable encodings of human domain knowledge. Such ontologies are used in several different ways [2-4]. Firstly, they ensure standardisation of terminology and identification across all entities in a domain so that multiple sources of data can be aggregated through comparable reference terms. Secondly, they provide hierarchical organisation so that such aggregation can be performed at different levels for novel data-driven scientific discovery. Thirdly, they facilitate browsing and searching in an easily accessible fashion. They also allow for logic-based intelligent applications that are able to perform complex reasoning tasks such as checking for errors and inconsistencies and deriving logical inferences. Logic-based knowledge representation (where ontologies serve as knowledge engineering artefacts) can be contrasted with algorithmic 'knowledge representation', in which software algorithms procedurally define outputs based on stated inputs, and with statistical 'knowledge representation', in which complex statistical models are trained to produce outputs based on a given set of inputs by learning weights for a complex set of internal parameters. An advantage of logic-based knowledge representation is that it allows the knowledge to be explicitly expressed *as knowledge*, i.e. as statements that are comprehensible, true and self-contained, and available for modification by persons without a computational background such as domain experts; this is in contrast to statistical methods that operate as black boxes and to procedural methods that require a programmer in order to manipulate or extend them.

Bio-ontologies have enjoyed increasing success in addressing the large-scale data integration requirement emerging from the recent increase in data volume [4]. One example of such a successful bio-ontology is the Gene Ontology (GO) [5], which is used *inter alia *to unify annotations between disparate biological databases and for the statistical analysis of large-scale genetic data to identify genes that are significantly enriched for specific functions. For the domain of biologically interesting chemistry, the Chemical Entities of Biological Interest ontology (ChEBI) [6] provides a classification of chemical entities such as atoms, molecules and ions. ChEBI organises chemical entities according to shared structural features, for example, carboxylic acids are all molecular entities that possess the characteristic carboxy group, and according to their activities in biological and chemical contexts, for example, acting as an antiviral agent. ChEBI is widely used as a database of chemical entities that can be queried both by structural classes and by functional annotations in the role ontology. The ontology has been applied in diverse applications such as annotation of chemicals in biological databases for pathways, interactions, and systems biology models [7-9]; chemical text mining [10]; formalising the chemistry underlying biological ontologies [11]; semantic similarity [12]; and metabolome prediction [13].

With the large-scale availability of chemical data through projects such as PubChem [14], making sense of the data and mapping between different internal and external collections has become one of the most pressing challenges facing chemical integration into modern biomedical science. Such mappings are facilitated by the spiderweb of annotations and cross-references attached to each entity in a chemical ontology such as ChEBI: the mappings to other chemical identifiers (such as InChI, PubChem, KEGG, DrugBank, Chembl, Reaxys and, where publicly available, CAS), and the annotations that use the ontology identifiers to identify chemical entities in biological databases such as pathway databases, protein interaction databases, systems biology modeling databases, biochemical reaction databases and many more. The availability of such a growing dictionary of cross-references in the public domain that operates at a broader level than only that of fully-specified chemical structures(as InChI does) allows mapping to be extended to classes of chemical entities that may behave similarly and therefore be described in one reference in a reaction database, for example.

Similarly to GO, ChEBI is manually maintained by a team of expert curators. Historically, bio-ontologies such as GO and ChEBI have been developed as Directed Acyclic Graphs (DAGs), a deliberately simplified ontology format which allowed domain experts (non-logicians) to directly participate in ontology engineering at a time when tools that supported more sophisticated semantics were rather difficult for non-technical persons to use. However, with the increasing availability of supporting tools and widespread adoption, there is a growing trend of evolution of bio-ontologies towards the greater expressive power provided by the Web Ontology Language (OWL) [15] and its extensions, which provides a sophisticated suite of logic-based constructs to support eloquent knowledge representation and automated reasoning in real-world domains [16]. ChEBI is an ideal ontology to take advantage of increasing formalisation, due to the elegant inherent regularities and symmetries in the chemical domain.

However, there has been little communication between the logicians driving the research underlying ontology technology and applications and the computer scientists and cheminformaticians driving the more traditional chemistry data management approaches. Thus, the applicability of the approaches commonly used in cheminformatics and in logic-based ontology, and potential interactions between these approaches has not heretofore been systematically assessed with respect to the requirements in the chemistry domain. It is to address this gap that we offer the current communication, addressing the following issues:

1. We present the requirements for structure-based chemical classification based on the results of an analysis of the structure-based classes included in the ChEBI chemical ontology;

2. We map the features identified in our requirements analysis onto the capabilities of existing cheminformatics tools for class definition and hierarchy construction, and to available logical formalisms underlying ontology technology;

3. We describe several strategies for combining these different methods to achieve a hybrid approach that harnesses the strengths of each field while meeting the challenges common to both;

4. We identify open research areas in structure-based chemical classification and ontology.

We anticipate that this will facilitate research both in the primary area of logic-based reasoning that underlies ontology technology and in cheminformatics, and pave the way for fruitful cheminformatician-logician collaborative opportunities. We further aim to facilitate the enhancement of the representation of chemical knowledge throughout biomedicine with accompanying benefits in disciplines such as drug discovery, metabolomics, systems biology and chemical genomics.

The remainder of this paper is organised as follows. This Background section presents some relevant chemistry classification and ontology preliminaries. Following that, in our Results we firstly present the types of classes used in chemical classification and thereafter compare these types of classes to the capabilities of hierarchy construction methods in cheminformatics and those of logic-based methods in chemical ontologies. In our Discussion, we further elucidate the relationship between cheminformatics and logical approaches, and present some applications of chemical ontology. We conclude with our outlook and open research areas.

### Classification in chemistry

The ability to *classify *raw information into meaningful groups is an essential component of human intelligence, which thus far has proven difficult to replicate in machine reasoning, except in narrowly defined domains. In particular, classification has a long tradition in chemistry: the periodic table of the elements is one of the longest-standing and most-used systems of hierarchical classification throughout the natural sciences.

The type of hierarchical classification that interests us here is *structure-based *in that it is the classification of molecules into groups based on which atoms in them are connected to which other atoms or aspects of overall atomic constitution. In both chemical synthesis in the lab and biosynthesis in organisms, the methods and pathways involved are entirely based on related structures, and this is why so many research agendas and publications in chemistry involve *classes *of chemicals, examples of which are:

• Synthesis of (pyrazolo)pyrimidines/pyridines

• BOP-mediated one-pot synthesis of *C_5_*-symmetric macrocyclic pyridone pentamers

• Halonium-initiated electrophilic cascades of 1-alkenoylcyclopropane carboxamides: efficient access to dihydrofuropyridinones and 3(2*H*)-furanones

• Spontaneous formation of a dibromoborenium cation driven by interaction between a borane Lewis acid and an arene *π *system

• Structural diversity for phosphine complexes of stibenium and stibinidenium cations

or involve a natural product based name such as a 'polyketide' or a 'spongistatin'.

This categorisation of chemical space is orthogonal to the sorts of machine-learning based classification traditionally used in cheminformatics analyses that concentrate on whether a molecule is likely to bind to a particular site in a protein or to display a particular activity based on a heuristic analysis of large amounts of data. These types of classification are not relevant for the methods described in the current paper, although the methods of classification we describe here are frequently used to delineate the input for training these sorts of classification methods.

### Benefits of classification in chemistry

The benefits of classification systems are severalfold. Classification *organises *large volumes of information into sensible groupings so that they are more accessible to humans. Such hierarchical organisations can be more easily browsed; research in cognitive science shows that humans can only browse and compare a relatively low number of concepts at the same level at the same time, thus grouping into hierarchies reduces the amount of detail that has to be dealt with at each level [17]. A hierarchical structure allows narrowing in on the area of interest within a large domain, and only exploring the details of that narrowed in area, rather than observing the full domain at such a detailed level. A second benefit of a hierarchical organisation is that it allows for the compact representation of generalised knowledge at the highest level to which it applies. For example, statements that are true for all mammals need to be expressed at the level of mammals as a whole, and not repeated for every specific mammal that occurs. Similarly, features that apply to all carboxylic acids can be expressed at the level of carboxylic acids as a whole, rather than repeated at the level of the different molecules as is required in databases or other flat structures that allow no general grouping or hierarchical organisation.

Hierarchical organisation of knowledge in a domain allows for data-driven discovery, enabling useful predictions to be made. For example, in functional genomics, the analysis of large-scale genetic data is facilitated by the grouping together of different genes that perform the same function. Modular analysis of such data reveals organisation at an aggregate level which is sometimes not apparent at the level of the raw data, due to overloading of detail and noise in the underlying signal. Hierarchical organisation of knowledge also allows useful predictions to be made, since it allows generalisation of knowledge to the highest possible level of applicability, and consequent prediction of properties of novel discovered members of the class.

Chemical classes, the objects found within a chemical classification system, group together chemical entities in a meaningful, scientifically relevant hierarchy. Ideally, all members of a chemical class should share important causal powers, such as undergoing decarboxylation in common circumstances. In fact, almost the only methods of classification available to historical chemists, before compound structures were well understood, were (i) based on the observation of reactivity through means of performing controlled reactions between different substances; or (ii) based on the origin of the molecule, when the molecule was isolated from a natural product substance. Much of these historical forms of classification are still inherited today, and are taught in chemistry classes and reproduced in textbooks. Knowledge about the structural features that form the underlying causes of the shared dispositional properties (where such existed), and the structural features shared between similar natural product substances, was only developed later. However, now that chemical structures are well described (within the limits of the chemical graph formalism), many more structural features are able to be used for chemical class definitions.

Note that in this paper, we do not attempt to compare hierarchical classification approaches with non-classification-based approaches to large-scale data management. Such an endeavor would be very valuable, but is out of scope for our current contribution. Rather, we assume the context of hierarchical classification systems that are already in use within the communities using chemical data, and in that context we will compare different approaches to representation and automation.

### Structure-based and non-structure-based classes

Interesting classes in chemistry can be grouped into those which are structure-based and those which are not. Structure-based classes are defined based on the presence of some shared structural feature across all members of the class. This feature, however, may be crisply defined or vaguely defined. Crisply defined structural classes will form the focus in this paper, and are discussed further in the section *sec:resultsclasses *below. Vaguely defined structural classes, by comparison, are those based on a family resemblance between a group of molecules, that are often of natural origin or have biological relevance. For example, *steroids *are defined in ChEBI as 'Any of naturally occurring compounds and synthetic analogues, based on the *cyclopenta[a]phenanthrene carbon skeleton*, partially or completely hydrogenated; there are usually *methyl groups *at C-10 and C-13, and often an *alkyl group *at C-17. By extension, one or more bond scissions, ring expansions and/or ring contractions of the skeleton may have occurred.' The vagueness is indicated by terms and phrases such as 'usually', 'one or more' and 'may have'. The approaches to chemical class definition that we will discuss in this paper are not able to represent such vagueness, although extensions such as fuzzy logic or logic enhanced with probability constraints may in the future be able to support this use case.

Chemical classes can also be defined based on where the chemical came from in synthetic or natural pathways. Chemicals of natural metabolic origin are called *natural products*. As our ability to determine molecular structure by such methods as crystallography, NMR, CASE has improved over the past century, so too has our ability to describe what is in a particular structural class. For example the klymollins [18], extracted from the coral *Klyxum molle*, are all produced by reactions from a common core molecule and have very similar connectivities and compositions. This is a common pattern for recently-discovered natural product molecules. Contrast this with alkaloids, one of the earliest classes of natural products to be identified, for which the best formal definition we have for the class reads (from ChEBI) 'Any of basic nitrogen compounds (mostly heterocyclic) occurring mostly in the plant kingdom (but not excluding those of animal origin). Amino acids, peptides, proteins, nucleotides, nucleic acids, amino sugars and antibiotics are not normally regarded as alkaloids. By extension, certain neutral compounds biogenetically related to basic alkaloids are included.' A flexible and expressive language is needed to fully do justice to the wide range of class names that are intuitive to chemists and can be found in natural language in electronic lab notebooks (such as are used in industry) and indeed in more traditional scientific publications.

Many interesting classes of chemicals are defined based on what the chemical does (its function or activity) in a biological or chemical context. Included in this group are drug usage classes such as antidepressant and antifungal; chemical reactivity classes such as solvent, acid and base; and biological activities such as hormone [19]. These are included in ChEBI under the 'role' ontology.

While the standardised descriptions of bioactivity assays and experimental protocols in chemical discovery are out of scope for our discussion in this paper, we note briefly that other projects within the chemical biology community are addressing these needs, including the BioAssay Ontology [20] and the Ontology for Biomedical Investigations [21].

Hybrid classes are composed from an intersection of the members of two different classes, howsoever defined. Examples are 'tricyclic antidepressant', 'tetracyclic antibiotic', 'organofluorine pesticide', 'pyrazole pesticide', 'organophosphorus pesticide' and 'thiourea pesticide'. Compositional entities such as these are easily dealt with by logical intersection, described further below. Throughout this paper, we operate on the assumption of compositionality, which is the notion that the meaning of the whole is completely determined by the meanings of the parts and the way that they are arranged. If there were compounds that were tricyclic and antidepressant but were not themselves 'tricyclic antidepressants', these would be violations of compositionality, because then there would be some extra condition not present in the name which would be necessary to decide whether something was itself a 'tricyclic antidepressant'. Compositionality works in chemistry and is harnessed in name-to-structure software such as Opsin [22], but for hybrid classes in which some aspects of the class definition is not structural, a database of annotated chemicals to non-structural classes is needed, as is provided by ChEBI.

### Desiderata for structure-based classification

The desiderata that we identify for structure-based classification in chemistry are as follows:

1. Class definitions should be expressed in a language or formalism which is accessible to domain experts (chemists);

2. It should be possible to *combine *different elementary features into sophisticated class definitions using compositionality;

3. The specification of class definitions should allow *automatic arrangement *of those classes into a hierarchy, i.e. it should not be necessary to manually place classes into a hierarchy as is currently done in ChEBI;

4. Mid-level groupings within the constructed hierarchy should be semantic, i.e. they should make sense to chemists and be *named*;

5. It should be possible for the system to automatically classify compounds (based on a description of their structural features) within the *most specific classes *to which they belong.

A further benefit of a formalisation of class definitions is that this would allow disambiguation of different class definitions that are used by different communities in reference to the same entities. For example, some communities may use the term 'hydrocarbons' as encompassing derivatives such as chlorohydrocarbons, while other communities may use the term in a stricter sense. The use of different definitions for the same class may lead to different chemical hierarchies as produced by classification tools implementing the same algorithms (structure-based and/or logic-based). Standardisation of class definitions across disparate communities requires communication between cheminformaticians/logicians and chemists. Formalisation of class definitions in support of automatic classification allows explicit disambiguation of these different senses; this can be achieved through convergence on a community-wide shared ontology which assigns different labels to classes that are defined differently, but which provides both of the disputed versions of the definition, thus allowing different user communities of user to select their preferred version.

### Ontological knowledge and logic-based reasoning

Logic lies at the heart of modern knowledge representation (KR) technologies. Logic-based representation employs formal methods developed in the context of mathematical logic in order to encode knowledge about the world. The key advantage of these methods is that the knowledge is stored in a machine-processable form. A core feature that the vast majority of KR formalisms share is the use of a well-defined syntax and semantics. The syntax serves as the alphabet of the language: it provides a set of symbols and a set of rules that regulate the arrangement of the symbols in valid expressions. The semantics enriches the syntactic objects with a meaning so that expressions complying with certain syntactic forms, known as axioms, have a universal and predefined interpretation. It is their semantics that enables machine processing. A set of valid syntactic expressions, known as *axioms*, constitutes an *ontology *in the computer science sense.

The amenability of KR languages to automated reasoning is of crucial importance. A reasoning algorithm - relying on principles of logical deduction - detects possible inconsistencies and computes the inferences that follow from a set of formally defined axioms; note that a reasoning algorithm is tied uniquely to the specific syntax and semantics of the given KR language. A reasoning engine can be used to check the logical consistency of a set of logical axioms. For instance, if a knowledge base (i) defines organic and inorganic compounds as disjoint chemical classes (ii) contains the fact that cobalamin is an organic compound and (iii) also classifies cobalamin as inorganic, then a contradiction will be detected. Another standard reasoning task is the discovery of information that is not explicitly stated in the ontology. For example, if an ontology categorises cobalamin as a B vitamin and also asserts that B vitamins participate in cell metabolism, then the fact that cobalamin participates in cell metabolism is derived. The automation of the above tasks - traditionally performed by humans - has a clear advantage as it permits the allocation of research resources to more intellectually demanding activities.

A reasoning procedure needs to exhibit certain properties in order to be practically useful. Namely, a reasoning algorithm needs to derive *correct *inferences, that is inferences that are in accordance with the semantics of the language; this property is known as *soundness*. Additionally a reasoning algorithm ought to be *complete*, i.e. to compute *all *the correct inferences that are entailed by a set of axioms. Finally, an essential requirement for a reasoning algorithm is to *terminate*, that is to issue an answer after a finite amount of time. A vital contribution of logic is that it can offer guarantees - by means of formal proofs - for the soundness, completeness and termination of a reasoning algorithm for *all *input ontologies. A KR formalism for which a sound, complete and terminating reasoning algorithm exists is (informally) called *decidable*, though strictly speaking and according to the formal definitions of logic, it is the problem of deciding whether a knowledge base is inconsistent that is (un)decidable, rather than the actual language. As a consequence, decidability is a highly desirable feature for a logic-based formalism that is suitable for being the foundation of real-world applications.

Apart from decidability, another important feature of KR formalisms is tractability, that is how expensive the reasoning tasks are in terms of computational resources, e.g. performance time. The trade-off between the expressive power and the tractability of a logic-based language is a fundamental one: increasing the expressivity of the language usually results in a more resource-consuming reasoning algorithm or even undecidability. For instance, consider first-order logic (FOL) and propositional logic (PL); FOL allows one to model a much broader range of statements than PL. For example, FOL allows to encode that for every molecule X, if × is organic and contains a hydroxy group, then × is an alcohol, whereas in PL one may state that implication only for *one specific *molecule. Nevertheless, reasoning in propositional logic is decidable, whereas reasoning tasks in unrestrained first-order logic are undecidable.

The need for decidable formalisms has been the driving force behind the development of Description Logics (DLs), a family of logic-based languages with well-understood computational properties and rich expressivity. DLs serve as the underlying formalism for the Web Ontology Language (OWL).

A powerful feature of OWL is the ability to perform *automatic classification *using highly optimised OWL reasoners. For instance, given the following axioms (illustrated in Manchester OWL syntax [23]):

(1) ZincAtomsubclassOfMetalAtom

(2) MetallicCompoundequivalentToCompoundandhasAtomsomeMetalAtom

(3) ZincOxidesubclassOfCompoundandhasAtomsomeZincAtom

An OWL reasoner can automatically infer by (1)-(3) that ZincOxide is a subclass of Met MetallicCompound. OWL is extensively used for knowledge representation and reasoning purposes in the Semantic Web. While, in general, OWL is a very efficient KR formalism for the encoding of tree-like structures (i.e. those whose 'branches' do not rejoin), it is fundamentally unable to correctly represent cyclic structures, such as molecular entities containing rings [24]. OWL exhibits the tree-model property [25] that on the one hand ensures important computational properties, such as decidability, but on the other hand prevents the users from describing non-tree-like structures using OWL axioms. For instance, one may state using OWL axioms that cyclobutane has four carbon atoms, but it is not possible to specify that these four atoms are arranged in a ring. Therefore, one of the prevailing challenges in chemical knowledge representation is crafting logic-based formalisms that are able to faithfully represent cyclic structures and, thus, support ontology-based applications that automatically classify chemical compounds.

## Results

### Analysis of structural features used in class definitions

By examination of the definitions of higher-level structural classes included in ChEBI, we have identified the following categories of elementary features used in structural chemical class definitions:

1. Interesting parts (IP), such as the carboxy group or the cholestane scaffold

2. Basic chemical properties (CP), such as the charge of the entire species

3. Topological features (TF), such as rings, chains and fused ring systems

4. Mechanical connectivity and shape (MC), such as rotaxanes, host-guest compounds, catenanes and cage compounds

5. Schemata for structural formulae (SF) such as C*_n_*H_2*n*_.

Most of these elements can be used singly or in combination with other elements via compositionality. Further explanations as well as examples follow in the sections below. For clarity, the classes and examples are summarised in Table 1, where each feature is assigned a unique code that will be used in the sections that follow.

**Table 1:** A summary of the features used to define structure-based classes, either singly or in combination

**Abbrev**.	Feature	Description	Examples of features	Examples of classes
IP.1	Skeleton	The main carbon backbone of the molecule	A porphyrin skeleton	Porphyrins, pyridines

IP.2	Attached group	A functional group attached in some position on a skeleton	A methyl group on a pyridine skeleton	Methylpyridine

IP.3	Arbitrary part	A group or atom present in any position within the molecule	A carboxy group, an oxygen atom	Carboxylic acid, oxygen molecular entity

IP.4	Count of parts	The specific number, or a constraint on the number, of parts of a specified type	Three carboxy groups	Tricarboxylic acid

IP.5	Relative arrangement of parts	Relative arrangement of parts of a specified type	Relative arrangement of hydroxy group and amino group	Allothreonine, threonine

CP.1	Basic chemical properties such as charge	Presence of a specific number of charges, or unpaired electrons	Presence of a single positive charge	Anion, cation, dication

TF.1	Topological features - presence of cycles	Whether a molecule contains cycles of specified types	Presence of a cycle containing a hetero atom	Heterocyclic molecule

TF.2	Topological features - count of cycles	Presence of the specified number of distinct (smallest) cycles	Presence of two cycles	Bicyclic molecule, tricyclic molecule

TF.3	Topological features - interrelation between cycles (fusing, arrangements)	Relative arrangements and fusing between cycles	Presence of two cycles sharing one bond	Ortho-fused molecule

TF.4	Topological features	Overall aspects of connectivity, such as ring arrangements	Arrangement of rings into a cage shape	Polycyclic cage, fullerene, nanotube

MC.1	Mechanical connectivity	Mechanical connectivity/interlocking	Molecule with interlinked rotating parts	Rotaxanes, catenanes

MC.2	Mechanical shape of molecule	Features of overall shape of molecule	Knot-shaped molecule	Molecular Möbius strips, molecular knots

SF.1	Structural formula - atomic	A schema for structural formulae in terms of the overall atomic constitution	Molecule with formula C*_n_*H_2*n*_	Hydrocarbons, alkanes
SF.2	Structural formula - repeating substructural units (polymers)	Structural formulae in terms of relative numbers of substructural units	Macromolecule with repeated ethylene units	poly(ethylene), poly(propylene)

### Interesting parts (IP)

Perhaps the most prominent of methods for classifying chemical entities based on features of their structures is based on the presence or absence of specific parts. Such parts may be the overall 'skeleton' of the structure or they may be minor constituents. The skeleton is usually loosely defined as the major or most relevant part of the molecule, the 'backbone' to which other groups are attached as decorations. For example, 'metalloporphyrin' is defined as any compound containing a porphyrin skeleton and a metal atom.

Note that as the term is commonly used in chemistry, a skeleton is not always a straightforward substructure, since bonds may be added or removed while retaining the same skeleton with different degrees of saturation. Allowing for different degrees of saturation, or the addition or removal of parts of the skeleton, gives rise to a vague class definition. Here, therefore, we focus on the stronger sense of skeleton that implies that the skeleton as specified must be a substructure of the molecule of which it is a skeleton. Classes defined with skeletons in this fashion are often named for the skeleton, such as 'porphyrin' for the compound and the class 'porphyrins'. Indeed, the same name is often used to mean a single compound, a class of compounds with the skeleton of that compound, and the larger class of compounds containing a part which has that skeleton [26].

Parts may also be straightforward constituents in which there is no implication that the part is somehow maximal, as there is in the case of skeletons. General parts are termed 'groups'. Groups may be simple atoms, and classes defined based on the presence of certain types of atoms can be organised according to the layout of the periodic table. Examples are 'carbon molecular entities' and 'lanthanoid molecular entities'. Classes qualify as subclasses of carbon molecular entities if they contain any atom of carbon, regardless of what other atoms they contain in addition. Classes qualify as subclasses of lanthanoid molecular entities if they contain any of the lanthanoid group atoms. As most complex molecular entities belong to several such classes, automation of this aspect of classification is obviously highly desirable. Groups may also be more complex, such as the carboxy group or the chloroacetyl group.

The number (count, cardinality) of such groups is also important. For example, tricarboxylic acids can be defined as a compound containing exactly three, i.e. no fewer and no more than three carboxy groups. With regard to the cardinality of groups within a molecule, a challenge that is absolutely key to machine-based classification in chemistry is *scalar implicature*. Scalar implicature means that when one specifies a number, that number is the maximal description of the number of entities of interest. While it is literally true that I have one leg, normal behaviour is to say that I have two legs, as this is maximally descriptive. The chemical parallel is as follows: if one is working in material science or developing liquid crystals, or are interested in lipids in biological systems, one will make extensive use of alkyl chains which are chains of methylene (CH_2_) groups. It is trivially true that a chain of *n *methylene groups is also a chain of (*n-1*) methylene groups. However, it would be misleading to describe a molecule with a dodecyl group attached as a methylated compound simply because it contains a substructure with the formula CH_3 _at the end of the alkyl chain.

The class definition may also specify the position at which a group (or set of groups) is attached to a skeleton. Such positions are assigned by rules for numbering the skeleton of a molecule in a reproducible (and community-agreed-upon) fashion.

Some particularly problematic classes refer to the relative arrangement of parts or attachments within the whole molecule. A special case is the relative configuration of stereocentres. Chemical graphs can be specified for completely stereochemically specified entities, and for completely stereochemically unspecified entities, but relative configurations of stereogenic centers cannot be specified using traditional chemical graph representation formalisms. For example, 'allothreonine' [*rel*-(2xtitR,3*R*)-2-amino-3-hydroxybutanoic acid] and 'threonine' [*rel*-(2*R*,3*S*)-2-amino-3-hydroxybutanoic acid] are compounds with a relative configuration of stereogenic centres, thus for which a graph cannot currently be drawn. What cannot be represented in the graph formalism is a relative arrangement of these: if one is up, the other is down or if one is down, the other is down. Another example are *gem*-diols, which are diols, i.e. compounds with exactly two hydroxy groups, where both hydroxy groups are attached to the *same atom*. Similarly, *α,β*-unsaturated alcohols have a double bond between the atom bearing the hydroxy group (the *α *atom) and one of its immediate neighbours (the *β*) atom. In the same fashion, *α,ω*-disubstituted compounds have substituents of interest to the chemist at either end of the molecule, regardless of its length.

### Basic chemical properties (CP)

Straightforward chemical properties such as charge and number of unpaired electrons are used to define broad classes of molecules such as ion and radical. The latter are particularly of interest to chemists working in the gas-phase, especially in atmospheric chemistry, where hydroxyl radicals play an important role in mopping up air pollution and can even be smelt at certain times of day. Aromaticity and saturation are other properties commonly found in class definitions. These also apply at a lower level of classification, such as 'aromatic diazonium ion'.

While aromaticity as a property is commonly algorithmically determined based on alternating patterns of single and double bonds within ring structures, we should note that there are edge cases for which aromaticity may not necessarily be safely inferred given a particular substructure. This is particularly true for large or heavily substituted systems [27].

### Topological features (TF)

Another element commonly used in class definitions is the number and arrangement of rings (cycles) in a ring system that is a part of the molecule. For example, the classes 'ring assembly' and 'polycyclic cage' both refer, in their definitions, to numbers and arrangements of rings in the molecule. Polycyclic cages are molecules that are composed entirely of cycles that are fused together in such a way as to form an overall cage-like structure. Examples are the fullerenes, cucurbiturils (so named for their similarity to pumpkins), nanotubes, and small regular compounds such as cubane. Polycyclic compounds are also named for the number of rings they contain, e.g. tetracyclic or pentacyclic.

The manner in which the ring systems is arranged may also have relevance. For example, an *ortho- and peri-fused *compound is a polycyclic compound in which one ring contains two, and only two, atoms in common with each of two or more rings of a contiguous series of rings. Such compounds have *n *common faces and fewer than *2n *common atoms; an *ortho-fused *compound is a polycyclic compound in which two rings have two, and only two, adjacent atoms in common, having *n *common faces and *2n *common atoms.

Related to the chemical properties from the previous section, the 'cyclic' modifier is often treated as an overall property of the molecule and as a modifier for other class types. Consider: 'cyclic ketone', 'cyclic peptide', 'cyclic ether' and 'cyclic tetrapyrrole'.

### Mechanical connectivity and shape (MC)

With the rising development within the field of nanotechnology and the development of molecular machines with the goal of emulating the performance and scale of biological machinery, chemists have been increasingly interested in molecules which are able to display device-like properties, including the presence of stationary and movable parts, and the ability to respond with controlled movements to the external environment. Classes of molecules that are mechanically interlocked - such as bistable rotaxanes and catenanes as well as pseudorotaxanes - are some of the most intriguing systems in this area because of their capacity to respond to stimuli with controlled mechanical movements of one part of the molecule (e.g. one interlocked ring component) with respect to the other stationary part [28]. Similarly, molecules which display unusual energetic properties by virtue of their overall shape, such as molecular Möbius strips and trefoil knots, are an active research area for many novel applications, and in many cases mimic the extraordinary properties of biomolecular machinery such as active sites within protein complexes [29,30].

### Structural formulae (SF)

Another form of definition by atomic composition is the definition of classes of molecular entity based on specifying the *exclusive *atomic composition. This can be contrasted to parthood (where other attachments are allowed). An example of such a definition is that for the class 'hydrocarbon', compounds that may contain only hydrogen and carbon atoms as parts. Note that the term 'hydrocarbon' is sometimes used ambiguously in chemistry between this strict sense and a broader sense in which molecules *derived from *hydrocarbon are also named hydrocarbons. An example of the latter is the class 'chlorohydrocarbon'. In this case, the relationship that is captured in ChEBI to 'hydrocarbon' is not 'is a' but 'has parent hydride', indicating the distinction between true and derived hydrocarbons.

Finally, an interesting, yet problematic to depict with existing graph-based tools, feature used in chemical class definitions, is that based on schemata for structural formulae. For example, 'alkane' is defined as 'an acyclic branched or unbranched hydrocarbon having the general formula C*_n_*H_2*n*+2_.'

This is similar to the scenario for defining macromolecules (from which polymers are composed), for example 'poly(ethylene)' that has the schematic formula (C_2_H_4_)*_n_*. Note that such macromolecules can be named (and classified) based on the individual source molecules from which the macromolecule was formed (usually through a polymerization chemical reaction) or from the resulting constitution subsequently to the chemical reaction taking place; this is known as source-based or structure-based naming respectively.

### Algorithmic and statistical approaches to automatic hierarchy construction

Cheminformatics solutions have been developed to classify sets of chemical entities automatically, both to search for robust relationships between structures and given biological activities and to organise large collections of data. Such algorithmic automatic classification systems are in common use in industry, particularly in areas such as drug discovery, agrochemicals and consumer goods.

Algorithms for automated classification tend not to perform efficiently when executed on arbitrary graph-based data structures, so a usual technique is to reduce graphs to characteristic *features *or *descriptors*, which serve as the input for classifiers. As defined by Todeschini and Consonni [31], a molecular descriptor is the final result of a logical and mathematical procedure that transforms chemical information encoded within a symbolic representation of a molecule into a useful number (calculated descriptors), or the result of standardized experiments (experimental descriptors). Among the calculated descriptors, if we focus on structural features, molecular fingerprints are binary strings in which each bit represents a feature. In the most common types of fingerprint, a feature could be either a pre-defined substructure or a random substructure mapped by a hashing algorithm.

**Figure 1:**
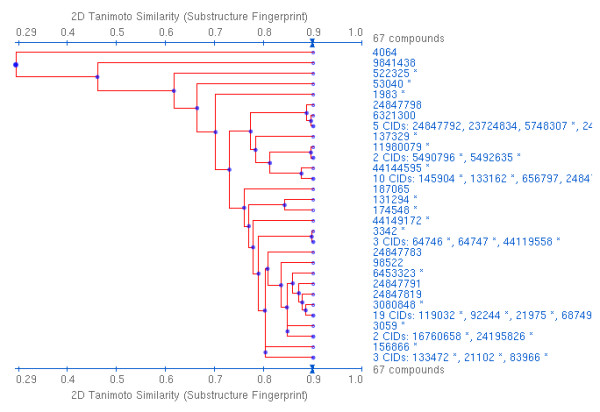
**Similarity-based hierarchical structure clustering**. Similarity-based hierarchical structure clustering is illustrated as it is computed in PubChem [14]. The figure was generated by searching for 'aspirin' and then executing the 'Structure Clustering' tool from the menu at the right. Numbers on the right are compound identifiers, unique numbers associated with chemical structures within the PubChem database.

In hierarchy construction algorithms, such as *hierarchical similarity clustering *[32,33], feature sets are clustered on the basis of high mutual pairwise similarity along a particular dimension. The clustering can be based on either agglomerative methods, where all instances are assigned their own class and these classes are merged, or divisive methods, where everything is assigned to a single class and this class is subdivided. The results depend both on the feature-identification algorithms and on the similarity calculations between resulting feature sets. There are many different algorithms for computing similarity measures between compounds and for aggregating compounds into clusters based on pairwise similarity measures, leading to arbitrarily many different classification hierarchies, even given the same compound collection as input. A limitation of such an analysis is that each node in the hierarchy represents a class with an arbitrary meaning, lacking a formal definition. The tree is highly sensitive to changes in input structures and the calculated features, making it very difficult to compare results across datasets even when the same clustering and fingerprinting algorithms are used. Another limitation is that most feature sets on which similarity measures are calculated, in order to be efficiently computable, represent only a subset of the total features of the molecules concerned, and local paths through the structures predominate over the (more expensive) overall molecular structure. Thus, molecules may turn out to appear quite similar according to such an algorithm (due, perhaps, to a predominance of similar parts), while displaying rather different structures overall. This problem is exacerbated for molecules of high structural regularity (e.g. polycyclic carbon compounds). Nevertheless, similarity landscapes are of paramount importance in reducing the complexity and understanding the features of large collections of compounds. Figure 1 shows an example of a similarity hierarchy generated by a similarity clustering tool that is part of the PubChem toolkit [14]. PubChem offers several different types of similarity clustering feature based on different underlying measures for similarity calculation, including two-dimensional and three-dimensional similarity. In terms of the features we identify for chemical classification, similarity-based hierarchy generation corresponds to the features used in the underlying fingerprint, which may be based on parts (IP) or chemical properties (CP), although the approach does not provide a generic solution that is able to handle all parts and properties, but is specific to those that are encoded in the underlying fingerprint.

While the above is mainly rule-based, machine learning approaches have become prominent in recent research. Supervised methods, such as Bayesian classifiers, decision trees and support vector machines, are employed to classify compounds for a particular functional activity class. However, these approaches result in binary output for non-structure-based classes. Supervised machine learning for prediction of chemical class membership based on an existing structural hierarchy is an interesting option, but would require large training sets of chemicals that are already classified. Although existing databases like ChEBI and MeSH [34] could act as training sets, the size of these data is still a tiny fraction of the enormous chemical space, and the problem is further complicated by the fact that the leaf nodes of such classification trees normally contain few structures. Manually constructed classifications may furthermore be far from complete in the sense that an arbitrary compound belongs to a vast number of classes yet will only have been classified under one or two - those deemed to be the most relevant.

**Figure 2:**
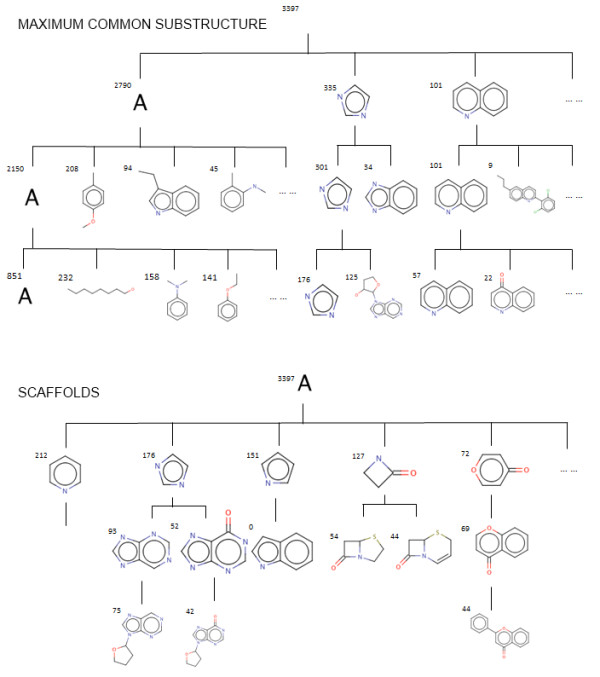
**Scaffold and MCS-based hierarchies**. Scaffold-based and maximum common substructure-based hierarchies are constructed by searching for shared common parts between a group of molecules. Higher positions in the hierarchy correspond to smaller shared scaffolds and substructures, with the root being 'any atom'. The MCS-based hierarchy includes non-ring structures, while the scaffold-based hierarchy only includes ring structures. Both images were generated based on hierarchies constructed using the structures belonging to the 'organic heterocyclic molecule' class in ChEBI.

Beyond feature-based, similarity-based and statistical approaches to automatic classification, an additional approach is classification based on substructures [35]. A substructure represents a wholly contained part of a molecule, and characteristic molecular substructures (skeletons or scaffolds and attached groups) are usually highly correlated with characteristic activities. Nodes in hierarchies based on substructures are able to be labelled with the relevant substructure that is shared for all members of the class; thus, such classes are more meaningful to humans than statistical or similarity-based classes. Variants on this approach include Maximum Common Substructure (MCS) based clustering and scaffold tree clustering. LibraryMCS [36] is a commercial application that can perform MCS based clustering on a set of structures. Although the technical details of the underlying implementation are not available, from the output it can be determined that structures sharing a common substructure are organized in the same class, and the common substructures define the scope of each class. Scaffold Tree [37] is a product that hierarchically classifies scaffolds, which are molecular frameworks obtained by removing side chains. By recursive removal of rings in scaffolds, scaffolds are decomposed into smaller ones which form the higher levels in the hierarchy tree. Along similar lines is the Scaffold Explorer tool which allows visualisation and interaction with scaffold hierarchies [38]. Chemical Abstracts Service (CAS) [39] offers a SubScape tool for visualisation and browsing based on scaffolds. Figure 2 illustrates an example of chemical hierarchies generated by scaffold and MCS approaches.

Both MCS- and scaffold-based methods allow visualisation and present an overview of a given dataset. Furthermore, the intermediate nodes which represent shared scaffolds or MCS structures correspond to the definition of classes based on an important part. These definitions could be extracted and formalised. But the output of these algorithmic approaches is still highly dependent on the input and thus could not act as a universal chemical classification system. (Even if it were possible to guarantee that the input corresponded to the universal chemical space, it is likely that the consequence would be a non-terminating classification algorithm.) These approaches are generally directly useful, since scaffolds often specify the general overall structure of the molecule, which in bioactive and especially in synthetic chemistry has a large influence on the activity of the molecule in the biological system. However, hierarchies based on scaffolding do not allow for the specification of overall properties of the molecule, nor for clustering based on similar aspects of molecules aside from their scaffolds. Scaffolds resemble skeletons, and MCS- and scaffold-based approaches address the automatic construction of hierarchies for classes defined based on interesting parts of the molecule (IP) with the exclusion of positional attachments (IP.2) and specific counts of parts (IP.4).

Leaving aside cheminformatics methods which have already been applied to automated hierarchy construction, there are other methods that have been used for definition of classes of molecules. A useful approach to the definition of chemical classes used in cheminformatics is the SMiles ARbitrary Target Specification (SMARTS) [40], that allows the specification in a compact line notation of structure-based classes of chemicals. SMARTS allows the expression of features that members of a class must have, including features such as atom types, bond types, cycles and aromaticity. An example SMARTS for the class of aliphatic amines is [$([NH2][CX4]),$([NH]([CX4])[CX4]),$[NX3]([CX4])([CX4])[CX4])]. Wild cards are supported, as are logical operators such as 'and'. SMARTS is a rich language for specifying structure-based chemical classes. Until lately, it was not very well supported by visualisation and editing tools, but a graphical displayer for SMARTS was recently released [41], and various structure editors provide support for SMARTS editing, including the PubChem chemical structure editor [42], although without yet making use of the SMARTS Viewer visualization for generic features. The PubChem chemical structure editor allows specification of SMARTS atom environments using the GUI query interface, and these are visualised by annotation on the atom in the rendered chemical structure and converted into SMARTS codes that can be used in PubChem searching. PubChem also makes use of SMARTS in defining features of molecules used for aspects of the error detection, standardisation and fingerprinting procedures in the PubChem computational architecture. Limitations of SMARTS are that it does not provide support for repeated units such as duplicated attached groups or an aliphatic carbon chain within a range of length, and the support provided for logical operators is limited in applicability to atoms, bonds, or nested features using the recursive group definition option. SMARTS are not compositional in the general sense, as substituents need to be enumerated explicitly.

SMARTS can be compared to query formalisms such as the Markush structure encoding formalism commonly used in patents [43] and the Molecular Query Language [44]. The molecular query language (MQL) provides a context-free grammar for description of parts of molecules, including primitives for atoms, bonds, properties, branching, and rings. Markush structures allow the description of compound classes by generic notation around the chemical graph formalism. The core of the representation is the specification of a compound scaffold together with varying parts. Types of varying parts that can be specified include R-groups, link nodes, atom lists, position variation and repeating units with repetition ranges. Such query languages facilitate matching against compound collections and they provide a compact representation that can serve as input to combinatorial enumeration algorithms. However, query formalisms do not lend themselves straightforwardly to generic computation of the arrangement of classes into a hierarchy, although it would of course be possible to write dedicated algorithms which performed such arrangement based on a specified set of definitions in any of the formalisms. The computation of hierarchical organisation in a generic (domain-independent, i.e. not specific to chemistry) fashion is one of the key benefits of logic-based ontology technology, as well-studied reasoning algorithms allow the rapid computation of hierarchical arrangement of large sets of class definitions as well as the computation of the most specific class to which a given compound belongs based on its structural features.

Another approach within algorithmic cheminformatics that is closely related to the hierarchical classification of entities within the chemical domain is that of computing systematic names for structures and structures from names. IUPAC naming rules for compounds such as described in the 'Gold book' [45] and implemented in various tools including the open source Opsin [22] provide a method for obtaining a systematic name from a given chemical structure, and for interpreting a name to determine the intended underlying structure. Importantly, rules for chemical naming in IUPAC confer similar information to the classification of molecular entities into hierarchies in the sense that parts of a chemical name correspond to parts of the molecule, and the same parts of the molecule are also used for parts-based classification. Thus, there could be a close integration between software that computes names and software that computes classification. Such an integration would also allow the naming of mid-level groupings in a constructed hierarchy based on IUPAC rules. We are not aware of any research projects currently that combine these two approaches towards this goal. More importantly, however, we note that IUPAC rules generate systematic names, which can be unwieldy and lengthy, and that chemists in many cases prefer to use shorter trivial names such as 'caffeine'. Such trivial names cannot be automatically computed and need to be stored in a knowledge base such as ChEBI.

### Automatic classification in chemical ontologies

In this section we describe the applicability of several of the KR formalisms underlying ontology technology to structure-based class definition and classification, highlighting the capabilities and limitations of each formalism. The section is arranged according to the features outlined in the analysis of chemical class definitions.

### Interesting parts (IP)

Structure-based classification of chemicals based on the presence of specific functional groups is among the most well-developed areas of ontology-supported chemical classification. Existential quantification in OWL (expressed with keyword 'some') allows the definition of chemical classes based on the existence of parts. For instance, a compound is a carboxylic acid if and only if there *exists *a carbon contained in the compound such that (i) the carbon has a double bond with an oxygen and(ii) the carbon has a single bond with an oxygen that is connected through a single bond to a hydrogen (O = C - OH). This can be formulated in OWL as follows:

$$\begin{array}{c} \text{CarboxylicAcid}\;\text{equivalent}\;\text{hasAtom}\;\text{some}\;(\text{Carbon}\;\text{and}\;\left(\text{doubleBond}\;\text{some}\;\text{Oxygen}\right)\;\text{and}\\ \;\;\;\;\;\;\;\;\;\;\;\;\;\;\;\;\;\;\;\;\;\;\;\;\;\;\;\;\;\;\;\;\left(\text{singleBond}\;\text{some}\;\left(\text{Oxygen}\;\text{and}\;\left(\text{singleBond}\;\text{some}\;\text{Hydrogen}\right)\right)\right)) \end{array}$$

We can represent formic acid (HCOOH) with the following OWL axiom:

$$\begin{array}{c} \text{FormicAcid}\;\text{equivalentTo}\;\text{hasAtom}\;\text{some}(\text{Carbon}\;\text{and}\;\left(\text{doubleBond}\;\text{some}\;\text{Oxygen}\right)\;\text{and}\;(\;\text{singleBond}\;\text{some}\\ \;\;\;\;\;\;\;\;\;\;\;\;\;\;\;\;\;\;\;\;\;\;\;\;\;\;\;\;\;\;\;\;\;\;\;\;\;\text{Hydrogen})\;\text{and}\;\left(\text{singleBond}\;\text{some}\left(\text{Oxygen}\;\text{and}\;\left(\text{singleBond}\;\text{some}\;\text{Hydrogen}\right)\right)\right)) \end{array}$$

By performing OWL reasoning we correctly infer that FormicAcid subclassOf CarboxylicAcid. However, according to this definition for formic acid there is no restriction on *additional atoms *that an instance of formic acid may contain; i.e. it is acceptable if an instance of formic acid contains an additional nitrogen. This is due to the open world semantics underlying OWL, and creates obvious problems for the correct representation of fully specified chemical structures such as formic acid that are not intended to allow additional atoms; it thus prohibits the definition of chemical classes based on the absence of some kinds of atoms. Additionally, as OWL cannot faithfully describe non-tree-like structures, this approach is not applicable to structure of functional groups with rings, such as phenyl groups.

One of the first uses of OWL for chemical classification was by Dumontier *et al*. [46], who classified molecules based on the presence of functional groups into an OWL ontology where the functional groups were described by axioms similar to the above. The tree-model property of OWL is acknowledged as a restriction, and DL-safe rules [46] are recommended as an alternative, although in fact the use of DL-safe rules for this purpose is also limited, as we discuss in the below section *Topological features*. More recently, this work has been extended in the Lipid Ontology, which encodes classes of lipids using OWL axioms for automatic classification of lipids [47]; the classification is mostly dependent on the detection of specific functional groups which is done using algorithmic approaches. This work has been recently extended towards classification of chemicals in ChEBI and MeSH in [48], which includes an algorithm for the discovery of shared features among groups of chemical structures and the assertion of those features into an OWL ontology. The features which are detected again include common functional groups and additionally the presence of charges and cycles.

In related work extending the notion of parthood and features for classification of chemical entities, Stevens describes an approach using OWL for the classification of the atoms in the periodic table [49].

### Basic chemical properties (CP)

A number of chemical classes are specified using numerical features of chemical entities, such as charge or mass. OWL 2 offers facilities for advanced handling of datatypes such as integers or strings. Datatypes allow knowledge engineers to define classes by referring to particular values or value ranges: for example, one may define *small *molecules as the molecules whose molecular weight is less than 800 Daltons. Furthermore, OWL 2 reasoners provide datatype reasoning support [50] in order to exploit this knowledge and derive new inferences: if it is stated that the weight of atropine is 289 Daltons, then atropine is automatically classified as a small molecule.

This is a convenient feature in applications such as e.g. drug discovery, that require filtering out molecules above a critical weight. For instance, one may want to retrieve all the small tetrapyrrole molecules, that is compounds that contain four pyrrole rings and with weight less than a threshold value.

### Topological features (TF)

One of the first attempts to overcome the limitations of OWL for representing cycles was DL-safe rules [51]. The extension of OWL ontologies with DL-safe rules allowed certain reasoning tasks to be performed over non-tree-like structures while preserving decidability. Nevertheless, the restrictions that are necessary in order to enforce decidability restrain the applicability of the rules to only explicitly named objects of the ontology - that is, individuals. Assuming a simplified knowledge base whose contents appear in Table 2 (bonds are assumed to have been defined as symmetric), an inference engine can derive the assertion CyclicMolecule(m) but not that Benzene subclassOf CyclicMolecule, as the DL-safe rules extension does not allow the deduction of subclass relationships that concern *all *the benzene molecules.

**Table 2:** An example of rules that are safe for use with OWL ontologies

OWL axiom	hasAtom some RingAtom subclassOf CyclicMolecule
OWL assertions	Benzene(m),singleBond(a_1_,a_2_), doubleBond(a_2_,a_3_), singleBond(a_3_,a_4_), doubleBond(a_4_,a_5_), singleBond(a_5_,a_6_), doubleBond(a_6_,a_1_), Carbon(a_i_),hasAtom(m,a_i_) for each 1≤i≤6

DL-safe rule	^1≤*i*≤6 Carbon(x_i_)^singleBond(x_1, _x_2_)^ doubleBond(x_2_,x_3_)^singleBond(x_3_,x_4_)^

	doubleBond(x_4_,x_5_)^ singleBond(x_5_,x_6_)^ doubleBond(x_6_,x_1_)→RingAtom(x_1_)

In order to address the need for class-level reasoning over structured objects as outlined above, a further OWL extension was suggested that combines OWL, rules and *Description Graphs *(DGs), a new modelling primitive for the representation of complex structures [52]. Using unextended OWL, a benzene ring is modelled with the following OWL axiom, which states that an object is a benzene ring if and only if it has exactly six carbon atoms each of which has a single bond with exactly one carbon atom and a double bond with exactly one carbon atom:

$$\begin{array}{c} \mathrm{BenzeneRing}\;\mathrm{equivalentTo}\;\mathrm{hasAtomexactly}6(\mathrm{Carbon}\;\mathrm{and}\;\mathrm{hasSingleBondWith}\;\mathrm{exactly}\;1\;\mathrm{Carbon}\\ \;\;\;\;\;\;\;\;\;\;\;\;\;\;\;\;\;\;\;\;\;\;\;\;\;\;\;\;\;\;\;\;\;\;\;\;\;\;\;\;\;\;\;\;\;\;\;\;\;\;\;\;\;\;\;\;\;\;\;\;\;\;\;\;\;\mathrm{and}\;\mathrm{hasDoubleBondWith}\;\mathrm{exactly}1\;\mathrm{Carbon}) \end{array}$$

Note that this representation would be different if aromaticity was explicitly included in the model. In that case, we would replace the single and double bond relationships with a single aromatic bond relationship.

**Figure 3:**
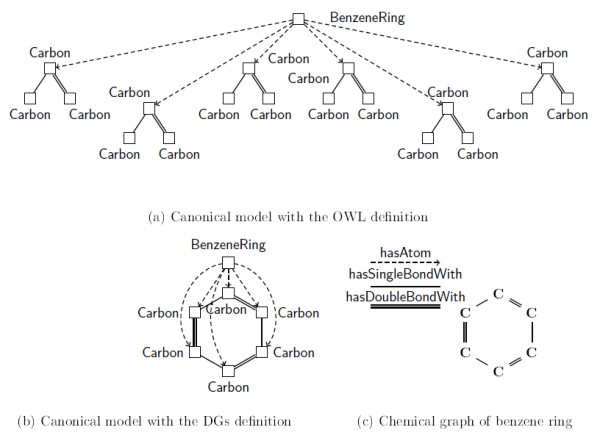
**Logical models of the benzene structure**. The chemical structure of benzene is illustrated together with the logical models of the class in the OWL language.

Figure 3 (a) shows the 'canonical' model of this benzene ring according to the OWL semantics: informally, the canonical model is what the logical definition encodes. The OWL model is tree-shaped.

Using DGs, the canonical model looks like Figure 3(b) that, in contrast to Figure 3(a), does capture the underlying cyclic structure.

However, certain syntactic restrictions are enforced on knowledge bases containing DGs in order to guarantee decidability. One of these restrictions is the *strong separation requirement *that prevents the user from mixing properties used in the OWL ontology with properties used in the DGs axioms. Therefore, if one uses the property hasSingleBondWith in the DGs formulas, then hasSingleBondWith may not occur in e.g. an axiom of the form hasSingleBondWith subPropertyOf hasBondWith. This constraint imposes limitations on the applicability of the formalism to general utility for chemical ontology, as evaluated in [53].

In an effort to relax the limitations imposed by the DGs approach, a radically different KR formalism with the name *Description Graph Logic Programs *(DGLP) has been developed [24]. The DGLP framework adopts the logic programming paradigm in order to represent objects whose parts are interconnected in arbitrary ways. Unlike description logics, the decidability guarantees of logic programs do not rely on the tree-model property and, so, the modeller is no longer restricted to tree-like structures. Since DGLP ensures decidability in different ways, the need for strong property separation is eliminated; thus, the ontology designer is free to mix up properties for both structured objects and general knowledge of the domain which implies more flexibility in the modelling decisions.

To represent classes with more advanced overall topological features such as polycyclic cages is beyond the expressivity of DGLP as it requires quantification over all atoms in a molecule rather than specific parts or properties. An approach for the representation of the overall structure of highly symmetrical polycyclic molecules is set out in [54] using a combination of monadic second-order logic and ordinary OWL. This approach has not yet been implemented in practice, but shows promise for logical reasoning over features involving regularity in the overall structure of molecules.

### Structural formulae

Some chemical classes are defined in part by the absence of certain characteristics, such as e.g. hydrocarbons (strictly defined, excluding derivatives), in which atoms of types other than hydrogens and carbons are absent. Inorganic molecules are often defined as those that do not include carbon atoms. Note that some carbon-containing molecules, such as carbonates and cyanides, are often classified as inorganic carbon compounds. These exceptions would need to be appended as additional constraints on the definition. However, we leave this complication aside in what follows.

Due to the open world semantics of OWL, everything that is not explicitly stated in the ontology is assumed to be *not known to hold *rather than *known not to hold*. This property of the semantics is a challenge for the knowledge engineer in capturing conditions based on the absence of information. For instance, consider the following OWL representation of a water molecule:

(4) WatersubclassOf(hasAtomexactly1Oxygen)and(hasAtomexactly2Hydrogen)

Consider also the following OWL definition of inorganic molecules:

(5) hasAtomonly(notCarbon)subclassOfInorganic

In accordance with the OWL semantics, Water subClassOf Inorganic is not derivable as there are models of water that comply with axiom (4) but contain additional carbon atoms. One may eliminate these models by constraining the number of atoms that water may contain:

(6) WatersubclassOf(hasAtomexactly3owl:Thing)

Nevertheless, Water subClassOf Inorganic is still not inferred as there are models of water that contain exactly three atoms (two hydrogens and one oxygen), but one of the three atoms is also classified as a carbon atom. One may overcome this difficulty by requiring the chemical elements to be disjoint:

(7) HydrogenandCarbonsubclassOfowl:Nothing

(8) OxygenandCarbonsubclassOfowl:Nothing

The axioms (4)-(8) do entail that Water subClassOf Inorganic. However, this is specific to this small and constrained knowledge base, and eliminating undesirable models by gradually adding axioms in this fashion is clearly a solution of little practical use, as it is domain-specific and tedious.

In contrast to OWL, logic programming is equipped with closed-world semantics; in the chemical domain context, this means that a molecule whose chemical graph is fully defined is presumed not to consist of any additional structure. DGLP [24] consequently has closed-world semantics and therefore allows the concise description of categories of molecules such as hydrocarbons or inorganic molecules. DGLP has been tested in practice for automatic classification of chemical molecules on classes such as hydrocarbons, inorganic molecules, molecules with a four-membered ring and molecules with exactly two carbons, with fair performance (for an ontology with 70 molecules, no test took more than a few minutes), which is expected to further improve with optimisation.

A category of molecules that is particularly challenging to represent with logic is the one defined by a parameterised molecular formula, such as alkenes which are described by the formula C_n_H_2n_. Constraints on number of atoms of particular sorts can be expressed using OWL cardinality restrictions, but this facility does not allow the relationship between the number of carbons and the number of hydrogens to be expressed.

The description of macromolecules such as polyethylene which consist of repeating units is also challenging. While the above formalisms can be used to describe the repeated units, the fact that the units are arranged in a chain is not easily described, and the fact that the number of repeated units is variable and not known beforehand cannot be straightforwardly encoded.

## Discussion

Historically, logic-based approaches to automated classification and cheminformatics approaches have developed largely independently. Our purpose here is to evaluate them side by side and compare and contrast their strengths and weaknesses.

The strength of algorithmic approaches used in cheminformatics is that they are able to be optimised and tweaked for the chemical domain and specific chemically relevant applications. However, there are nevertheless several key benefits to adoption of the logic-based ontology-driven approach in the chemistry domain, namely:

• Taxonomical knowledge represented in an ontology is *explicit *and accessible to domain experts, while algorithms which perform hierarchical classification often act as *black boxes*, and amending the classification methodology requires adapting the underlying software or re-training a complex statistical model.

• Using an ontology for classification allows for *explanations *(justifications) [55], both for computed subclass relations and for detected inconsistencies. This can be contrasted to black-box approaches such as neural networks where no explanation services are available.

• Representation of chemical knowledge in an ontology allows it to be harnessed in a generic fashion from within diverse ontology-based applications which also utilise knowledge from *other domains *(a core requirement for whole-scale systems biology), while to make use of cheminformatics algorithms and toolkits requires custom software, differing from the software used in other domains.

• There are several features needed for chemical class definition that are not adequately catered for in algorithmic approaches, but which can be formalised in logical expressions (although not always in straightforward OWL), such as the absence of atoms of a particular type, or features of regularity in the overall structure.

In contrast to the algorithmic hierarchy construction, chemical ontologies allow the specification of a hierarchy from the top down, in the sense that the features of chemical classes can be specified by experts, and the assignment of their members is based on these features, rather than being restricted by what algorithms for detecting similarity or substructures are able to detect. Creating such a hierarchy allows for the explicit representation of domain knowledge, which corresponds to the content of textbook chemistry and at the same time can be interlinked with research reports in the literature as well as large-scale databases of chemical compounds. Targeted development of novel compounds with desirable properties for therapeutics and other applications relies on extensive domain knowledge, currently to a great extent only human-accessible via textual scientific literature or verbal communication from mentor to student.

The explicit representation of knowledge in this fashion allows for the classification of edge cases (unusual classes) and cases which cannot be treated within the constraints of the available algorithmic tools. Statistical (machine-learning) approaches rely on the underlying quantification of features in the molecules - and features that are not common are less likely to be represented in resulting trained models. Similarity comparisons are vulnerable to the specification of features to be used in the quantification of similarity. Also, many of the features used are path-based, that is, they traverse combinatorially exhaustive paths through the molecule *up to a certain length*. It is difficult to capture overall features of the molecule with path-based approaches. However, some overall features of molecules, such as count of rings, are often added in to the features used in such classifications. Substructure detection is similarly unable to account for overall features of molecules. Ontology-based classification using logical definitions gives a flexibility in defining features, even very large ones, or ones that span over a small number of examples but are nevertheless important and would otherwise be lost in the long tail. An important thing is that the eventual classification (howsoever arrived at) is provably correct, i.e. includes no false statements.

Examples of edge classes which appear difficult to deal with in the cheminformatics approaches are:

1. organometallic compound, because the underlying physics of their bonding is not susceptible to the valence-bond approach

2. cyclic peptide, because the cycle in question is not an arbitrary attached ring, but a cycle of chained peptide links and hence not obviously detectable

3. fullerene, just because they contain a vast number of rings which can cause ring-detection algorithms to time out

Chemists regularly assign names to mid-level class of chemical entities for use in scientific communication and education, which machine-learned groupings may not be able to discover. This leads to the situation where it is not possible, for example, to group together all the literature describing that category of chemicals, despite the fact that chemists think and communicate regularly in terms of such categories. This can be compared to the scenario in chemistry education, where relevant groupings of chemical entities are often taught in chapter-specific units. Of key relevance is linking classes of chemicals to the reactions that can be used to synthesize them, such as those described in the Name Reaction Ontology (http://rxno.googlecode.com/).

Due to the heterogeneous nature of ontology classes (i.e. not restricted to chemical structures), ontology-based representation also allows the description of functional classes of chemical entities, as is done in the ChEBI role ontology, and the linking of those to relevant structural classes. This can be applied to retrieval of all structures for a given functional class, e.g. all odorant molecules, in order to do primary research in a particular domain, e.g. smell perception. Here, the primary purpose of the research might not be chemical in nature but rather into perception, thus making the implementation of a targeted chemical database a costly overhead; therefore having this sort of functional grouping available in broader chemical knowledge bases such as ChEBI is a large benefit.

Such functional groupings of structures are essential inputs to many cheminformatics approaches. If it is possible to group together all molecules which act against the same receptor, it is then possible to train predictive models based on this information. Research in the sciences often examines groupings of chemical entities which exhibit shared behaviour in order to understand more about the mechanisms underlying that behaviour. Having to extract the grouping that one is interested in manually from the database by doing a literature analysis in every case is a labour-intensive task, and it is one that should be centralised so as to free up the resources of researchers for focusing on their primary research. Importantly, this sort of information needs to be hierarchically organised, so that it is not repetitively described, and so that it can be grouped and clustered at different levels of aggregation depending on the needs of the individual researcher. For instance, for some research purposes one may be interested in the classification of all molecules which are odorants; for other purposes, one may be interested in only those which smell sweet or smell bitter.

For these reasons, ontology-based chemical taxonomies have a valid place alongside the other methods for chemical classification. On the other hand, there are several benefits to adopting cheminformatics tools within the ontology engineering process in the domain of chemistry, such as to benefit from the well-developed and rapid algorithms for detecting parthood between chemicals and for computing properties. This presents a challenge for tooling and for algorithm research, in that the logic-based ontology tools and algorithms need to work alongside and be integrated with cheminformatics tools and algorithms. While substructure detection can be efficiently done outside of the ontology framework, crude assertion of all detected substructure relationships between molecules in an ontology leads quickly to a combinatorial explosion of asserted parts and relationships [56]. Yet, logical methods for substructure detection are bound to be less efficient than dedicated algorithms. There is a need for future work to showcase hybrid approaches taking into consideration the strengths and weaknesses of both methodologies, with the balance between the different approaches being empirically determined to maximise the efficiency and applicability of the overall system.

In summary, we can consider the desiderata for a structure-based classification system that we have identified in the Introduction and compare to the approaches which we have evaluated above.

*1. Accessibility to domain experts*. While OWL and other logical formalisms are not easily comprehensible to non-logicians, they do at least contain human-legible definitions for classes which can be inspected, unlike 'black box' approaches.

*2. Support for compositionality to define classes based on combinations of elementary features*. Logical approaches provide explicit support for compositionality via fundamental logical operations such as AND and OR. However, cheminformatics automatic classification via fingerprints and substructures also provide implicit support for compositionality of the features used in the classification algorithm. But these approaches, with the exception of SMARTS, do not customarily provide support for explicit definition of classes.

*3. Automatic arrangement of classes into hierarchies based on their definitions*. Of the cheminformatics approaches, SMARTS gives the most explicit support for definition of classes. However, the weakness of SMARTS is that it does not allow for automatic arrangement of classes into hierarchies based on the definitions. Other cheminformatic approaches such as MCS do allow construction of hierarchies, but not definition of classes. Logic-based formalisms such as OWL provide explicit support for the automated arrangement of definitions into hierarchies using reasoners.

*4. Semantic, named mid-level groupings*. One of the weaknesses of cheminformatic hierarchy construction approaches is that the mid-level groupings which they provide in their hierarchies are not explictly named and often do not have meaning outside of the particular hierarchy, i.e., they are not associated with any semantics. Logic-based approaches, with their explicit focus on logical definitions at all levels, do meet this requirement.

*5. Structure-based automatic classification of compounds into classes*. This is, of course, the primary strength of cheminformatics structure-based hierarchy construction methods. However, it is also possible with logic-based methods, as long as the features encoded in the chemical structures are made accessible to the logical reasoner. This can be achieved either by encoding the chemical structure within the logical formalism (where this is supported by the expressivity of the formalism) or by using cheminformatics approaches to extract the features as a precursor to the logical reasoning.

## Conclusions

We have presented an analysis of the requirements and the current functionality of available implementations for structure-based chemical classification and chemical ontologies. It is our hope that this work will contribute to the future development of synergies between cheminformaticians and computer scientists interested in classification of complex structures. Future work will be to create a benchmark for the performance evaluation of the approaches we have described in this contribution, including the evaluation of the time vs. space complexity of algorithms against a standard set of definitions and a standardised compound collection.

Structure-based classification is essential to many applications of chemistry in modern science, driven by the need to manage large-scale data and to stay ahead of newly generated knowledge across many different research areas amid exploding quantities of primary literature. Such literature reports are often phrased in terms of classes of chemical entities rather than individual fully specified molecules. Furthermore, biological knowledge such as the actions of enzymes in biological pathways is often described in terms of whole classes rather than individual molecules. While cheminformatics methods are highly optimised for operating on chemical structures, logic-based ontology technology allows for explicit knowledge representation in a more targeted fashion. There is a need for the development of hybrid systems that interface between domain-independent ontology technology and chemistry-specific cheminformatics methods.

Aside from the integration of logic-based and cheminformatic methods, an additional open research area is in the representation and reasoning with those of the features which are used in structure-based classification that are not covered by any of the available technologies here surveyed, including the mechanical connectivity and shape of molecules, the relative arrangement of parts, interactions between cycles, and the specification of repeating units arranged in a particular way such as in polymers. There is also a need for the development of tools in the area of visual editing of chemical class definitions.

## Methods

### Defining features used in structure-based chemical class definitions

The list of features (Table 1 - Features used to define structure-based classes) was extracted from a manual inspection of (i) the textual definitions and (ii) the members associated with classes in the 'chemical entity' branch of the ChEBI ontology. The initial inspection was carried out by three of the authors and the resulting list of features was discussed among all of the authors.

Higher-level classes were identified as those members of the chemical entity ontology that (i) were not themselves defined by an InChI (the IUPAC canonical representation of the chemical structure designed for identification and disambiguation of chemical entities, [57]), since InChI can only be generated for fully specified structures, (ii) had descendants in the ontology, and (iii) had a textual definition. Some of these were discarded on inspection as being out of scope for this study, as discussed in the Introduction.

A sample list of the textual class definitions, together with their class IDs and names, that formed the input to this analysis, is included in Table 3. The full list of textual class definitions is accessible via the ChEBI database and web services, and can also be obtained from the authors on request.

**Table 3:** A representative sample from the list of ChEBI classes used in the analysis

ChEBI ID	Name	Definition
CHEBI:50860	organic molecular entity	A molecular entity that contains carbon.

CHEBI:50047	organic amino compound	A compound formally derived from ammonia by replacing one, two or three hydrogen atoms by organyl groups.

CHEBI:51690	enaminone	A compound containing a conjugated system of an amine, an alkene and a ketone.

CHEBI:33567	catecholamine	4-(2-Aminoethyl)pyrocatechol [4-(2-aminoethyl)benzene-1,2-diol] and derivatives formed by substitution.

CHEBI:33860	aromatic amine	An amine in which the amino group is linked directly to an aromatic system.

CHEBI:51349	polyamine macromolecule	A macromolecule composed of units connected by imino (−NR-) linkages.

CHEBI:51402	phenylenediamine	A benzene substituted with two amino groups.

CHEBI:59654	prolinols	The class of all compounds which contain a prolinol skeleton.

CHEBI:33709	amino acid	A carboxylic acid containing one or more amino groups.

CHEBI:60249	lead ion	A lead atom having a net electric charge.

CHEBI:58941	cyclic tetrapyrrole anion	An organic anion arising from deprotonation of a cyclic tetrapyrrole compound.

CHEBI:2580	unsaturated fatty acid anion	Any fatty acid anion containing at least one C-C unsaturated bond; formed by deprotonation of the carboxylic acid moiety.

CHEBI:58955	branched-chain fatty acid anion	Any fatty acid anion with a carbon side-chain or isopropyl termination.

CHEBI:33598	carbocyclic compound	A cyclic compound in which all of the ring members are carbon atoms.

CHEBI:33658	arene	Any monocyclic or polycyclic aromatic hydrocarbon.

CHEBI:33847	monocyclic arene	A monocyclic aromatic hydrocarbon.

CHEBI:38976	alkylbenzene	Benzene substituted with one or more alkyl groups.

CHEBI:35302	helicene	*ortho*-Fused polycyclic arenes in which all rings (minimum five) are angularly arranged so as to give helically shaped molecules.

CHEBI:51198	calixarene	A macrocycle composed of 1,3-phenylene groups linked by methylene groups. The number of 1,3-phenylene units in the macrocycle is denoted by the *n *in calix[*n*]arene name.

CHEBI:33612	polyhedrane	A polycyclic hydrocarbon of the (CH)*_n _*formula having a skeleton corresponding to the regular or semiregular geometrical solid.

CHEBI:36786	tetralins	Compounds containing a tetralin skeleton.

CHEBI:50961	rotaxane	A system in which at least one macrocycle encloses another, rod-like molecule (shaft) having end groups too large to pass through the ring opening, and thus holds the rod-like molecule in position without covalent bonding.

CHEBI:51269	acenes	Polycyclic aromatic hydrocarbons consisting of fused benzene rings in a rectilinear arrangement and their substitution derivatives.

CHEBI:51586	benzoins	Compounds containing a benzoin (2-hydroxy-1,2-diphenylethanone) skeleton.

CHEBI:51614	diarylmethane	Any compound containing two aryl groups connected by a single C atom.

### Generation of scaffold and MCS hierarchies

The ChEBI molecules classified beneath organic heterocyclic compounds (CHEBI:24532) were processed using Scaffold Tree [37] and ChemAxon's LibraryMCS [36]. The class contains 3397 entities with chemical structures, for each of which at least one ring system is present. The structures are very diverse, from simple structures like pyridines to complex natural product structures like indole alkaloids.

LibraryMCS was executed via its GUI interface. Only highly frequent scaffolds were selected manually for visualization. In Figure 2, the number of structures containing the MCS is annotated beside the structure. All 3397 structures have a MCS of 'A', which means any atom. Other larger MCSs were displayed as leaf nodes of root MCS 'A'. Some interesting MCSs such as imidazolidine were found, but uninteresting MCSs such as carbon chains also appeared. Even in the third layer, there were still 851 structures in class 'A', which means no interesting MCS was found for that group.

The lower part of Figure 2 illustrates the hierarchy generated by Scaffold Tree. Scaffolds were organised with respect to the number of rings. Scaffolds that appeared frequently were also selected for visualization. As scaffolds are generated on the basis of ring systems, a better hierarchy was generated compared to the MCS based method.

## Competing interests

The authors declare that they have no competing interests.

## Authors' contributions

JH and CS designed and coordinated the project. JH, LD and ME analysed the chemical class definitions to extract structure-based features. LD and CB provided the input for the section on algorithmic classification. DM, JH, RS and CB provided the input for the sections on ontologies and logic-based classification. All authors contributed to, and have read and approved, the final manuscript.
